# Supplementary material for: Spongy moths from Europe and Asia: Who could have higher invasion risk in North American?
Source: PLoS One. 2025 May 8;20(5):e0320598. doi: 10.1371/journal.pone.0320598 (PMC12061144; doi:10.1371/journal.pone.0320598)
Supplement: S2 Table — (DOCX) [file pone.0320598.s002.docx]

| predictors | NASM | ASM | ESM |
| --- | --- | --- | --- |
| Annual mean temperature | 0.051 | 0.166 | 0.054 |
| Mean diurnal range(mean of monthly(max temp-min temp)) | 0.008 | 0.042 | 0.070 |
| Isothermality(bio2/bio7)(×100) | 0.043 | 0.073 | 0.055 |
| Temperature seasonality (standard deviation ×100) | 0.059 | 0.056 | 0.101 |
| Max temperature of the warmest month | 0.050 | 0.071 | 0.033 |
| Min temperature of the coldest month | 0.035 | 0.054 | 0.047 |
| Temperature annual range(bio5-bio6) | 0.014 | 0.074 | 0.199 |
| Mean temperature of the wettest quarter | 0.012 | 0.016 | 0.006 |
| Mean temperature of the driest quarter | 0.007 | 0.039 | 0.006 |
| Mean temperature of the warmest quarter | 0.124 | 0.067 | 0.268 |
| Mean temperature of the coldest quarter | 0.049 | 0.064 | 0.057 |
| Annual precipitation | 0.326 | 0.048 | 0.007 |
| Precipitation of the wettest month | 0.054 | 0.085 | 0.008 |
| Precipitation of the driest month | 0.012 | 0.029 | 0.007 |
| Precipitation seasonality | 0.011 | 0.009 | 0.009 |
| Precipitation of the wettest quarter | 0.066 | 0.049 | 0.001 |
| Precipitation of the driest quarter | 0.027 | 0.028 | 0.007 |
| Precipitation of the warmest quarter | 0.045 | 0.025 | 0.004 |
| Precipitation of the coldest quarter  Fractions of cropland  Fractions of managed pasture  Fractions of forested primary land  Fractions of non-forested primary land  Fractions of rangeland  Fractions of potentially forested secondary land  Fractions of potentially non-forested secondary land  Fractions of urban  Aspect  Elevation  Slope | 0.047  0.007  0.003  0.003  0.001  0.019  0.002  0.001  0.001  0.004  0.033  0.004 | 0.008  0.061  0.014  0.052  0.017  0.018  0.018  0.031  0.008  0.003  0.004  0.012 | 0.011  0.039  0.026  0.047  0.005  0.004  0.008  0.014  0.043  0.001  0.012  0.008 |

S2 Table Importance scores of each predictor in the preliminary ecological niche models

Note: NASM: Spongy moths of North America; ASM: Spongy moths of Asia; ESM: Spongy moths of Europe.
