# Supplementary material for: Spongy moths from Europe and Asia: Who could have higher invasion risk in North American?
Source: PLoS One. 2025 May 8;20(5):e0320598. doi: 10.1371/journal.pone.0320598 (PMC12061144; doi:10.1371/journal.pone.0320598)
Supplement: S4 Table — (DOCX) [file pone.0320598.s004.docx]

S4 Table Comparisons of AUC and TSS between real and null ecological niche models.

| NASM | | | | ASM | | | ESM | | |
| --- | --- | --- | --- | --- | --- | --- | --- | --- | --- |
| Models | AUC | TSS | Models | AUC | TSS | Models | AUC | TSS |  |
| Real | | 0.995 | 0.938 | Real | 0.970 | 0.815 | Real | 0.930 0.923  0.928 0.924  0.927 0.815  0.806 0.805  0.817 0.812 | 0.699 0.686  0.691 0.685  0.693 0.532  0.490 0.516  0.541 0.533 |
| Real | | 0.996 | 0.942 | Real | 0.970 | 0.816 | Real |  |  |
| Real | | 0.996 | 0.944 | Real | 0.968 | 0.802 | Real |  |  |
| Real | | 0.995 | 0.941 | Real | 0.967 | 0.801 | Real |  |  |
| Real | | 0.995 | 0.939 | Real | 0.970 | 0.815 | Real |  |  |
| Null | | 0.881 | 0.641 | Null | 0.854 | 0.548 | Null |  |  |
| Null | | 0.861 | 0.584 | Null | 0.871 | 0.603 | Null |  |  |
| Null | | 0.867 | 0.606 | Null | 0.861 | 0.571 | Null |  |  |
| Null | | 0.867 | 0.635 | Null | 0.850 | 0.535 | Null |  |  |
| Null | | 0.863 | 0.606 | Null | 0.836 | 0.505 | Null |  |  |

Note: NASM: Spongy moths of North America; ASM: Spongy moths of Asia; ESM: Spongy moths of Europe.
