# Supplementary material for: Spongy moths from Europe and Asia: Who could have higher invasion risk in North American?
Source: PLoS One. 2025 May 8;20(5):e0320598. doi: 10.1371/journal.pone.0320598 (PMC12061144; doi:10.1371/journal.pone.0320598)
Supplement: Attachment — Submitted filename: Responses.docx [file pone.0320598.s006.docx]

**Responses to Reviewer #1**

Dear reviewer:

We sincerely appreciate your insightful comments and all the suggestions you provided for our manuscript, which have highlighted several shortcomings in our manuscript. Additionally, your profound comments on our topic demonstrate your expertise in the field, which we greatly admire. In our revised version, we have addressed all of your concerns. We have also provided detailed responses to the concerns you raised, which you can find in the following section of my point-by-point responses. Finally, we would like to express our gratitude for your diligent efforts in reviewing our manuscript. We hope that our revisions meet your expectations for the manuscript. Of course, if you have any further valuable suggestions, we sincerely welcome them, and we will make additional revisions based on your feedback.

Reviewer #1: Dear editor and authors, I saw this MS earlier submitted via another journal. My decision is same as previously, this MS could be published after revision according to recommendations provided below.

Submitted MS provide the data of comparing the constructed models of L. dispar expansion in North America in the case of different population origins (from Asia or from Europe). The results of this comparison are given with in MS. I think it is good study which touch actual problem of species invasion, especially using as the example the species belonging to top 100 dangerous invasive species in the word. I also think that this study is acceptable for the auditory of considering journal. My criticism is belong to put this study more deep in actual knowledge about systematics, genetics and ecology of studied species that would make more close of discussion perspectives to observing reality at the moment. I think after this improving this MS might be accepted by the journal. See deleted comments below.

**Our responses:** Thanks for your very professional comments. We have tried our best to revised our MS. If you have any further valuable suggestions, we sincerely welcome them, and we will make additional revisions.

L38-41 Please refer also to more modern studies like Picq et al., 2023 who study SM populations in worldwide format demonstrating that population structure is more difficult than thought earlier.

**Our responses:** Thanks for your very professional comments. We have added more recent studies to our revised MS at line of 41, including those by Picq et al. (2023). Thanks!

L 51-53. Sorry but I miss the logic of this part of text. L.dispar was invade in north America from Western Europe (i.e. by nonflight females) that was confirmed by genetic studies (Wu et al., 2015, Molecular ecology, Picq et al., 2023 Evolutionary application). Does author discuss here the potential of Asian strain invasion or recorded fact of Asian spongy moth invasion in last sentence? If the last, please justify.

**Our responses:** Thanks for your very constructive comments. We wrote this part of text for comparing the different flight ability of these two strains. However, with a reference to your following comment, it is not a key or suitable point. Therefore, we have removed it, and compared their invasion history and host ranges, which might induce their different invasion potentials at lines of 46-52.

L 54-57. Yes, this is logical prediction but it could miss with facts. For example, both strains have the features of ballooning of first instar larvae (Martemyanov et al., 2019, Plos ONE), that could be more important for spreading than modest flight ability of females which are nonfeeding and can not flight for a long time (I,e, 750m/day, 5-6 days maximum, i.e. the distance of migration is 4-5km per season). If you will compare the moving of border range in North America (studies by Sharov or Tobin) or in Asia (Ponomarev et al., 2023, Insects for example), in both cases the distance will overcome the maximal flight ability of females. So flight ability of Asian population is less likely critically important for the speed of expansion in continental scale. Please consider this augments in more detail.

**Our responses:** Thanks for your very constructive comments. We fully accept your professional comment, and removed this phrase. In our revisions, we compared their invasion history and host ranges which might induce their different invasion potentials in North America at lines of 46-52.

L75-82 When you discuss the effect of climate on L. dispar please put it in the context of microclimate. It is very important for such phenomenally adaptive species as LD and behavioral adaptation is one of the most important mechanism for such adaptation. For example formally supercooling point of L. dispar overwintered embryos is between -23-29C (Fält-Nardmann et al., 2018), however owing to laying the eggs masses under the snow cover (Ananko, Kolosov, 2021, Journal of Thermal Biology) or on the rocks in mounting areas (Ananko et al., 2022, Insects) that allow embryos avoid such temperature even outside temperature is significantly lower (-30C and lower). So overwinter temperature is unlikely to be an direct important climatic factor. I would say that microclimate condition are more limiting for LD expansion then general climate conditions. Please include this facts in the discussion of the theory.

**Our responses:** Thanks for your very constructive comments. We fully accept your professional comments. We have made revisions by saying that microclimate conditions are more limiting for LD expansion than general climate conditions at lines 73-82. Thanks!

L109-114 I think authors need to present this paragraph in more detail and more clear. Did I correct understand that you plan to use for created SDM the parameters from European and Asian populations of SM? This is important paragraph which will make more clear the following text.

**Our responses:** Thanks for your professional comments. We agree with you. We have rewritten this paragraph in more detail. In our study, we employed species distribution model tools to examine the potential range of the introduced spongy moths in North America. We also built SDMs for the native spongy moths in Asia and Europe, separately, and the models were generally determined by their traits or adaptation to the environmental conditions. Then, we transferred the models to North America, and projecting their potential ranges there, and the revisions could be found at lines of 117-127. Thanks!

L243 please decode MSS

**Our responses:** Thanks for your kind reminding. We have decoded MSS at line of 200.

Fig 2 please make the figure caption more informative and clear: which territory, which populations. Otherwise it sound confusing: c) “Range of native spongy moths in Europe” while figure present North American continent.

**Our responses:** Thanks for your very kind reminding. We have made revisions on Figure 2. Specifically, (a) Potential ranges of introduced spongy moths in North America. (b) Projected potential ranges in North America for the spongy moths from Asia. (c) Projected potential ranges in North America for the spongy moths from Europe. Thanks!

L 303-308 I agree that European SM and Asian SM possess wider range than American population but I think that for Asian SM the border is too wide and at south border (at least according to figure 1) include closely related species such as Lymantria obfuscata (India), L. xylina (China), L. albescens (Japan). Please check it.

**Our responses:** Thanks for your very rigorous comments and kind reminding, which push us to review and check our source occurrence data. From GBIF, we found that the southmost occurrence in Asia was in Sri Lanka (7.1N, 80.1E), from which the occurrence of our target species was detected on 10^th^ Aug 2021 (<https://www.gbif.org/occurrence/3466823587>). It, to certain extent, was supported by a study (https://doi.org/10.1038/s41598-019-57020-7) which argued that the suitability for this species was considered high in southern parts of India and Sri Lanka. We fully understand your professional inquiry. Maybe, a closely related species was mistakenly identified as *Lymantria dispar* Linnaeus. Also, maybe it truly is *Lymantria dispar* Linnaeus as a result of one of accidental events in the spreading of this species. However, the reliability of this occurrence record depends on the professional ability of the observer, and we have not solid evidence to definitely and easily deny its reliability. However, if you tend to remove it, we will delete it in our future revisions. Thanks again.

L 311-312 you could also argue that North American population went though strict bottleneck (Wu et al., 2015) that would decrease plasticity level of invader population,

**Our responses:** Thanks for your very professional comments. We fully agree with you, and have made revisions at lines of 341-344.

L 313-314. I am not native English speaking researcher but I think to avoid confusing between insects sources and range (territory) better to write “spongy moth FROM Asia/Europe” if I correct understand what authors want to say. If it is correct please use this correction for other parts of text whenever it was used.

**Our responses:** Thanks for your very kind reminding. Your comment clearly reflects what we want to say, and we fully agree with you. Accordingly, we have made relevant revisions through our MS, making it clearer and smoother. Thanks again!

L 313-320. I hope this data based on true L. dispar records (i.e. not on closely related species) with correct species identification. This question address to the data sources used for this study rather than to authors of this study. To me the south border of Asian SM range includes some closely related species. In turn, I did not find north Africa locations for European spongy moth populations (see Picq et al., 2023) what will make the latitude gradient equal for both strains Asian and European subspecies.

**Our responses:** Thanks for your very professional comments, and we fully agree with and understand your professional inquiry. The southmost occurrence of our target species in Asia was recorded in Sri Lanka (7.1N, 80.1E, 10^th^ Aug 2021, [www.gbif.org/occurrence/3466823587](http://www.gbif.org/occurrence/3466823587)), which, to certain extent, was supported by a study (<https://doi.org/10.1038/s41598-019-57020-7>) arguing that the suitability for this species was considered high in southern parts of India and Sri Lanka. However, we have to acknowledge that maybe, a closely related species was mistakenly identified as *Lymantria dispar* Linnaeus. Also, maybe it truly is *Lymantria dispar* Linnaeus as a result of one of accidental events in the spreading of this species. However, the reliability of this occurrence record depends on the professional ability of the observer, and we have not solid evidence to definitely and easily deny its reliability. However, if you tend to remove it, we will do it in our next revision version. Moreover, in our study, we included three populations of *Lymantria dispar* Linnaeus as our study objects, i.e., populations of *Lymantria dispar* from Asia, Europe and North America, and populations of *Lymantria dispar* from Asia and Europe could not necessarily equal to the Asian and European spongy moths, respectively. Therefore, in our study, European spongy moth populations North Africa was not incorporated into our study. We hope our explanation could satisfy you. If you have further inquiries on it, please don’t hesitate to tell us in future revisions. We appreciate your professional comments, and have revised our phrases to make it clearer.

L 322-327 I miss what authors would say here. You could use more informative reference (Djoumad et al., Scientific report) describing unique mt genome of Caucasus populations of Lymatria dispar which possibly will described as separate subspecies in future (see Picq et al., 2023). But anyway I do not understand what kind of comparison here authors would make. I confuse in the context of following text and miss what authors want to highlight. Please make it more clear and logic.

**Our responses:** Thanks for your very professional comments which make us reconsider the contribution of this paragraph to our topic. We agree with your comment that this paragraph seems irrelevant to our topic. Therefore, we have removed it from our revised MS. Thanks!

L 332-337. Again, maximal female flight ability of Asian strain measured in Km per season is significantly low to compare with speed of spreading of range border. For example see recent study of Ponomarev et al., 2023, Insects where speed of border range moving is 50 km/year and studied population belong to European spongy moth according to genotyping by sequencing markers (Picq et al., 2023). So female flight ability is mostly local tactics of the species rather than spreading strategy. Typical example is population in Altai mountings which formally belong to European genotypes, but the life cycle is obligatory related with female flight ability allowing to lay eggs on the rocks remote from host plant (Ananko at al., 2022, Insects). So this traditional features of Asian SM populations is less likely to seriously invest to spreading of population at continental scale.

**Our responses:** Thanks for your very professional comments, and we agree with you. Owing to your valuable comment, we realized that using female flight ability to explain the difference of range sizes between the populations of *Lymantria dispar* from Asia and Europe is misleading. Therefore, we have removed it from our revised MS. Thanks!

L380-389 As I read it I think that logic is broken here. If most data used in the analysis were collected in the part of the season this mean that stable range could be less then described according to the reasons described in this paragraph. Possibly I misunderstand the message of author, anyway this mean that rewording is needed.

**Our responses:** Thanks for your very professional comments, and we fully agree with you and have made revisions at lines of 420-424.

I think after major revision of this MS it could be acceptable for the journal.

**Responses to Reviewer #2**

Dear Reviewer:

We greatly appreciate your positive feedback on our manuscript, which is very exciting for us. Additionally, we are grateful for the valuable comments and suggestions you provided for our manuscript. Following your suggestions, we have carefully revised the content of the manuscript and thoroughly addressed your queries on certain sections. Please refer to the point-by-point responses for specific details.

Lastly, we sincerely thank you for your diligent efforts in reviewing our manuscript. We hope that the revisions made to the manuscript meet your expectations. However, if you have any further suggestions or valuable insights, we warmly welcome them. We will use your feedback as a basis for any necessary adjustments and improvements.

Reviewer #2: Major Comments:

Population Structure:

What is the population structure of spongy moths in North America? Does it represent a combination of European and Asian populations, or does it constitute a distinct, new population?

**Our responses:** Thanks for your very professional comments. According to the arguments by some scientists, population of spongy moths in North America is a combination of European and Asian populations. We have made revisions at lines of 130-131. Thanks!

Methods:

The description of the methods is unclear. Please provide additional details, specifically clarifying the purpose of each method and how it was implemented.

**Our responses:** Thanks for your very constructive comments, and we fully agree with you. We have added more detailed description for the methods.

Figures:

For Figure 2, there are 4 sub-figures, but the authors only explain 3. Similarly, Figure 3 contains 3 sub-figures, yet the authors describe only 2. Please ensure all sub-figures are adequately addressed in the text.

**Our responses:** Thanks for your very kind reminding. These are our mistakes. In Figure 2, 2-a was an unnecessary or redundant sub-figure, and in Figure 3 3-a was also an unnecessary or redundant sub-figure. They were subfigures in our previous versions of MS, representing the potential ranges of the spongy moths from Eurasia continent, and the range dynamics of the introduced spongy moths relative to those from Eurasia continent, respectively. We have made revisions on Figure 2 and Figure 3. Thanks again.

Minor Comments:

Typographical Errors:

Typos in lines 37, 43, 48, and 56 et al, need correction.

**Our responses:** Thanks for your very kind comment. We have checked and made revisions for typographical errors. Thanks again!

Line 111:

What does "SDMs" stand for? Please define the term for clarity.

**Our responses:** Thanks for your comment. We have defined the “SDMs”, i.e., species distribution models, at line of 119.

Line 157:

How were the importance scores of the remaining predictors calculated in the final species distribution models and the preliminary ecological niche models? Please elaborate.

**Our responses:** Thanks for your very professional comment. The important scores were calibrated through the Jackknife technique, and we elaborate it at lines of 177 and 201-202. Thanks!
